# Targeting Frequent Efferocytosis in Tumor Microenvironment is a New Direction for Cancer Treatment

**DOI:** 10.1002/smsc.202400479

**Published:** 2025-02-04

**Authors:** Gangxing Zhu, Xinliang Wan, Wanyin Wu, Luyu Jia, Xiaoyan Yu, Handan Mo, Xi Wang, Qichun Zhou, Qing Tang, Sumei Wang

**Affiliations:** ^1^ Clinical and Basic Research Team of TCM Prevention and Treatment of NSCLC Department of Oncology Guangdong Provincial Hospital of Chinese Medicine Chinese Medicine Guangdong Laboratory State Key Laboratory of Traditional Chinese Medicine Syndrome Guangdong Provincial Key Laboratory of Clinical Research on Traditional Chinese Medicine Syndrome State Key Laboratory of Dampness Syndrome of Chinese Medicine The Second Clinical College of Guangzhou University of Chinese Medicine Guangzhou 510120 Guangdong China

**Keywords:** apoptotic cells, cancer, efferocytosis, phagocytes, tumor microenvironment

## Abstract

A variety of factors, such as dietary habits, the external environment, and individual genetic differences, can lead to the development of cancer. While chemotherapy and radiotherapy are commonly used for cancer treatment, drug resistance and side effects are prevalent issues. Therefore, there is an urgent need to find new treatment modalities. Studies have shown that radiotherapy and chemotherapy can lead to a significant increase in apoptotic cells (ACs) within the tumor microenvironment (TME). The process of phagocytosis helps maintain homeostasis by engulfing and removing these ACs from the organism promptly, which is referred to as efferocytosis. However, it has been observed that excessive efferocytosis can promote the formation of an immunosuppressive TME, which is detrimental to tumor therapy. Thus, inhibiting efferocytosis to enhance the formation of an immune microenvironment shows promise as a treatment direction for tumors. As researchers gradually uncover the molecular mechanisms of efferocytosis, various small‐molecule inhibitors and monoclonal antibodies are actively being assessed in clinical trials. Targeting efferocytosis is anticipated to emerge as a promising approach in tumor treatment. In this review, the intricate steps involved in efferocytosis are explored and the current drugs that targeting this process for cancer treatment are outlined.

## Graphical abstract

Phagocytosis of “apoptotic tumor cells”, known as efferocytosis, accelerates the formation of an immunosuppressive microenvironment, leading to the detriment of tumor therapy. Blocking this process represents one of the novel approaches for treating tumors. Phagocytosis of apoptotic cells affects the efficacy of immunotherapy needs to be negated, whereas phagocytosis of tumor cells needs to be affirmed.

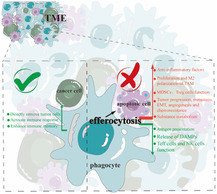

## 1 Introduction

The incidence of malignant tumors has remained high in recent years and stands as the leading cause of death among individuals under the age of 70. Recent research indicates that by 2020, there were an estimated 19.3 million new cases of cancer globally, with the global cancer burden projected to reach 28.4 million cases by 2040, representing a 47% increase from 2020.^[^ ^1^ ^]^ Among the available treatments for tumors, surgery may not be suitable for advanced patients and the elderly, chemotherapy‐based treatments can lead to drug resistance and various side effects, radiotherapy inevitably inflicts damage on the patient's body, and immune checkpoint inhibitors, the most prevalent treatment, are only effective for a subset of patients.^[^ ^2^, ^3^, ^4^, ^5^ ^]^ Hence, the quest for new therapeutic approaches is imperative.

**Figure 1:**
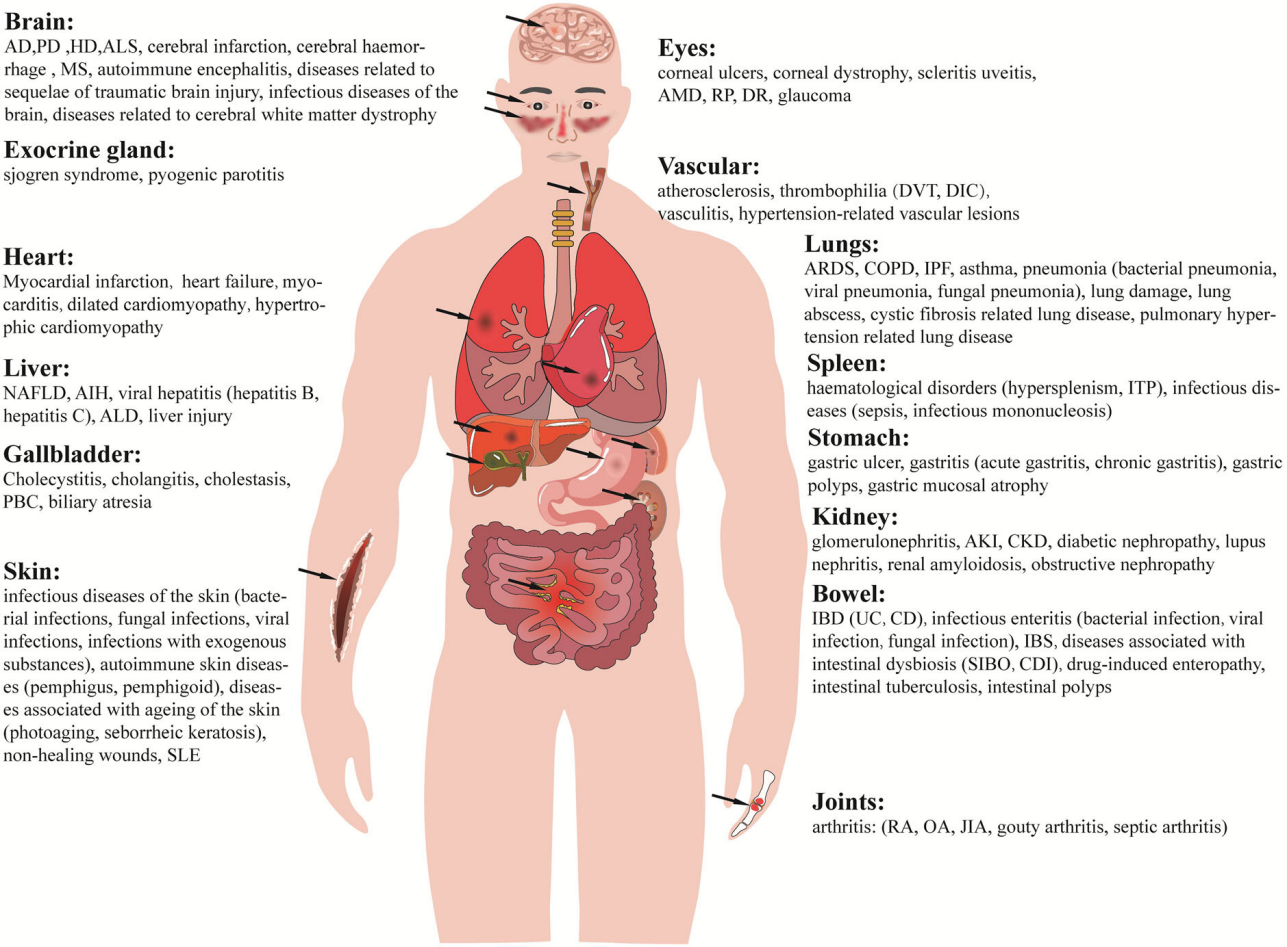
Efferocytosis disorders contribute to a spectrum of diseases. When phagocytes have a diminished phagocytic capacity, they struggle to eliminate ACs promptly, culminating in secondary necrosis and the subsequent release of cellular contents. This failure can disrupt the body's internal environmental equilibrium by influencing various intricate transcriptional programs and other regulatory mechanisms. Abbreviations: AD, Alzheimer's disease; PD, Parkinson's disease; HD, Huntington's disease; ALS, amyotrophic lateral sclerosis; MS, multiple sclerosis; NAFLD, non‐alcoholic fatty liver disease; AIH, autoimmune hepatitis; ALD, alcoholic liver disease; PBC, primary biliary cholangitis; SLE, systemic lupus erythematosus; AMD, age‐related macular degeneration; RP, retinitis pigmentosa; DR, diabetic retinopathy; DVT, deep vein thrombosis; DIC, disseminated intravascular coagulation; ARDS, acute respiratory distress syndrome; IPF, idiopathic pulmonary fibrosis; ITP, immune thrombocytopenia; AKI, acute kidney injury; CKD, chronic kidney disease; IBD, inflammatory bowel disease; UC, ulcerative colitis; CD, Crohn's disease; IBS, irritable bowel syndrome; SIBO, small intestinal bacterial overgrowth; CDI, clostridium difficile infection; RA, rheumatoid arthritis; OA, osteoarthritis; JIA, juvenile idiopathic arthritis.

Studies indicate that billions of apoptotic cells (ACs) are generated in the human body daily.^[^ ^6^ ^]^ Initially remaining intact, these cells eventually undergo secondary necrosis due to their inability to sustain plasma membrane integrity. This breakdown results in the release of intracellular contents and the production of numerous inflammatory cytokines, triggering inflammatory reactions and tissue damage.^[^ ^7^, ^8^ ^]^ Fortunately, the organism's abundance of phagocytes ensures the prompt and efficient removal of these ACs. Coined by Peter Henson and colleagues, the term “efferocytosis,” which translates to “carrying the corpse to the grave,” precisely describes the engulfment of ACs,^[^ ^9^ ^]^ predominantly executed by macrophages due to their heightened speed and capacity.^[^ ^10^ ^]^ Additionally, other specialized phagocytes (such as monocytes and immature dendritic cells), nonspecialized phagocytes (respiratory epithelial cells, intestinal epithelial cells, and fibroblasts), and tissue‐specific “specialized” phagocytes (like retinal pigmented epithelial cells, Sertoli cells of the testis, mammary epithelial cells, and fibroblasts) are all involved in this process.^[^ ^11^, ^12^ ^]^ Effective efferocytosis plays a vital role in maintaining homeostasis by averting the secondary necrosis of ACs, dismantling and expelling their cellular contents, releasing abundant anti‐inflammatory mediators, and governing intricate transcriptional programs.^[^ ^13^ ^]^ Efferocytosis disorders can contribute to various diseases, encompassing atherosclerosis,^[^ ^14^, ^15^ ^]^ systemic lupus erythematosus (SLE),^[^ ^16^ ^]^ rheumatoid arthritis (RA),^[^ ^17^ ^]^ chronic obstructive pulmonary disease (COPD), asthma,^[^ ^18^, ^19^, ^20^ ^]^ and numerous other diseases (**Figure** **1**).

The efficiency of efferocytosis is influenced by various factors, such as the ratio of phagocytes to the size of ACs and the type of phagocytosis (professional or nonprofessional).^[^ ^11^, ^21^ ^]^ The tumor microenvironment (TME) provides ideal conditions for high‐efficiency efferocytosis. First, within the TME, tumor‐associated macrophages (TAMs), professional phagocytes, account for over 50% of the tumor tissue and predominantly exhibit an M2‐type phenotype with robust efferocytosis capabilities.^[^ ^22^, ^23^, ^24^ ^]^ Second, tumors, particularly those postradiotherapy or with high‐grade malignancies, represent high‐turnover environments.^[^ ^25^, ^26^ ^]^ This heightened turnover not only increases the presence of TAMs and ACs but also intensifies their interactions. However, unlike in other diseases, efferocytosis within the TME is deemed a deleterious process due to several reasons: 1) the abundance of TAMs, inherently detrimental, exerts immunomodulatory functions across various aspects of tumor biology, spanning from initiation to progression, metastasis, epithelial‐mesenchymal transition (EMT), neoangiogenesis, and chemoresistance.^[^ ^27^, ^28^, ^29^, ^30^ ^]^ 2) Efferocytosis yields copious amounts of anti‐inflammatory cytokines and specialized pro‐resolving mediators, hastening the formation of an immunosuppressive microenvironment.^[^ ^29^, ^31^, ^32^ ^]^ 3) The release of anti‐inflammatory cytokines prompts TAMs to adopt an M2‐like phenotype with enhanced efferocytosis capabilities.^[^ ^33^, ^34^ ^]^ 4) Efferocytosis fosters the expansion and activation of myeloid‐derived suppressor cells, reinforcing tumor immune evasion by boosting regulatory T‐cells (Tregs) proliferation.^[^ ^35^ ^]^ 5) Efferocytosis diminishes the antigen presentation of tumor ACs, the release of damage‐associated molecular patterns, and subsequent immune responses.^[^ ^36^, ^37^ ^]^ 6) Efferocytosis promotes TAM proliferation, leading to the continuation of this malignant process in the TME.^[^ ^38^ ^]^ In summary, efferocytosis in the TME engenders a detrimental cycle of efferocytosis‐M2 polarization‐immunosuppression within the TME, ultimately facilitating tumor progression. Thus, inhibiting efferocytosis in the TME emerges as a promising avenue for tumor treatment.

This intricate yet sophisticated multistep process involves the recognition, phagocytosis, digestion, and breakdown of apoptotic cells by phagocytes. As research progresses, the molecular mechanisms of efferocytosis and its implications in cancer have become increasingly clear. Numerous drugs targeting different aspects of this process have been developed, demonstrating promising efficacy in impeding tumor advancement during preclinical stages and advancing to various clinical trials. In this article, we comprehensively outline the distinct stages of efferocytosis and provide a detailed overview of the corresponding drug categories. Subsequent sections delve into the specifics of each category.

## 2 “Find Me” Signaling Promotes TAM Recruitment around ACs

**Figure 2:**
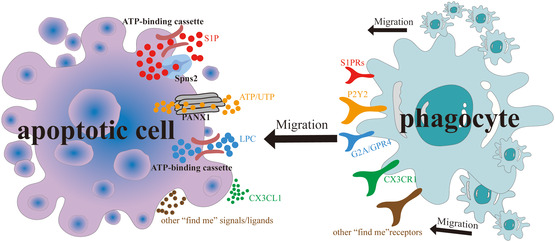
“Find me” signaling promotes TAM recruitment around ACs. Early apoptotic cells release various “find me” signals through multiple channels. Corresponding receptors on phagocytes detect these signals and migrate toward the ACs. This mechanism sets the stage for subsequent specific recognition and phagocytosis.

In *C. elegans*, the proximity of ACs to phagocytes facilitates their recognition and phagocytosis. Nevertheless, the considerable distance between ACs and phagocytes in mammals hinders the prompt clearance of remote ACs.^[^ ^39^, ^40^ ^]^ Fortunately, ACs can be drawn toward macrophages by secreting various cytokines, including proteins (e.g., C‐X3‐C motif chemokine ligand 1 (CX3CL1)), nucleotides (including adenosine triphosphate (ATP) and uridine triphosphate (UTP)), lipids (like lysophosphatidylcholine (LPC)), and lipid products (such as sphingosine 1‐phosphate (S1P)), etc. (**Figure** **2**). This long‐range attraction signaling ensures the efficient identification of distant ACs, leading to their subsequent recognition and phagocytosis.^[^ ^31^, ^41^ ^]^

### 2.1 S1P

Intracellular sphingomyelin, an omnipresent membrane lipid, undergoes metabolic catabolism to produce pro‐apoptotic ceramide. Ceramide can then be converted to sphingosine by the enzyme ceramidase, which is further phosphorylated to S1P by sphingosine kinase isoenzymes 1 and 2 (SphK1/2).^[^ ^42^ ^]^ Unfortunately, chemoradiotherapy induces an increase in ceramide levels, leading to the generation of a large number of apoptotic cells. This, in turn, upregulates the expression of SphK1 and SphK2, ultimately resulting in elevated S1P production.^[^ ^43^, ^44^ ^]^ S1P can be transported outside the cell with the assistance of ATP‐binding cassette or Spns2,^[^ ^45^, ^46^ ^]^ attracting macrophages through S1P‐specific G protein‐coupled receptors (S1PRs) on their surface.^[^ ^47^, ^48^, ^49^ ^]^ Significantly, this cascade activates the NFAT‐HIF1‐α signaling pathway, promoting autocrine‐mediated activation of the nuclear receptor peroxisome proliferator‐activated receptor‐gamma (PPAR‐γ) by erythropoietin (EPO). This process, on one hand, regulates the expression of inflammatory factors and drives M2‐like polarization, while on the other hand, it facilitates the transcription of numerous cytosolic molecules like Mertk, Gas6, MFGE8, and CD36, pivotal for the subsequent “eat me” stage.^[^ ^43^, ^50^ ^]^ Beyond the “find me” process mediated by S1P/S1PRs, S1P itself is recognized as a crucial oncogenic factor. Increased expression of S1P is observed in various malignant tumors such as breast cancer,^[^ ^51^ ^]^ gastric cancer,^[^ ^52^ ^]^ and pancreatic cancer,^[^ ^53^ ^]^ with its signaling playing a substantial role in cancer advancement. Therefore, targeting S1P emerges as a promising strategy for cancer treatment. Presently, a range of S1P signaling modulators have demonstrated promising results in tumor therapy (**Table** **1**).

**Table 1:** Targeting the “find me” phase.

Medicine	Type	Method	Disease	Stage	ClinicalTrials.gov identifier	Status	Mechanism	Source
Suramin	S1PRs antagonist	Combination with paclitaxel	Stage IIIB/IV metastatic breast cancer	I/II	NCT00054028	Completed, with results	1. Binds to SIPRs on the surface of phagocytes and prevents them from recognizing SIP released by apoptotic cells, thereby inhibiting the subsequent efferocytosis process. 2. Inhibited tumor invasiveness and metastasis 3. Inhibition of telomerase or fibroblast growth factors and angiogenesis cytostatic activity 4. Enhanced drug sensitivity	[220, 221, 222, 264, 265]
		Combination with docetaxel	Advanced NSCLC	II	NCT01671332	Completed, with results		
		Combination with paclitaxel and carboplatin	NSCLC	II	NCT00006929	Completed		
		Combination with docetaxel and/or gemcitabine	Stage IIIB/IV platinum‐refractory NSCLC	I	NCT00066768	Completed		
		Monotherapy	Bladder carcinoma	I	NCT00001381	Completed		
		Monotherapy	Hormone‐refractory prostate cancer	III	NCT00002723	Completed		
		Combination with leuprolide and flutamide	Untreated prostate carcinoma	II	NCT00001266	Completed		
		Combination with flutamide and hydrocortisone	Prostate cancer	III	NCT00002881	Completed		
		Combination with fluorouracil	Metastatic renal cell cancer	I/II	NCT00083109	Completed		
		Monotherapy	Recurrent bladder cancer	I	NCT00006476	Completed		
		Combination with radiation therapy (RT)	Newly‐diagnosed glioblastoma multiforme	II	NCT00004073	Completed		
		Combination with chemotherapy	Recurrent brain tumors	II	NCT00002639	Completed		
		Combination with doxorubicin	Advanced solid tumors	I	NCT00003038	Completed		
		Combination with RT and temozolomide	High‐grade glioma	I	NCT02490930	Completed		
Fingolimod (FTY720)	S1PRs modulator	Monotherapy	Progressed NSCLC or SCLC	II	NCT06424067	Not yet recruiting	1. Binds to SIPRs and perturbs downstream signaling pathways to regulate the expression of efferocytosis‐associated receptors. 2. Affects signaling molecules associated with phagolysosome formation in the digestive phase of efferocytosis and reduces its efficiency. 3. Inhibit tumor growth, angiogenesis 4. Improve T‐lymphocyte function	[223, 224]
		Monotherapy	Advanced solid tumors	I	NCT00661414	Completed		
Sonepcizumab (LT1009)	Anti‐S1P monoclonal antibodies	Monotherapy	Refractory RCC	IIa	NCT01762033	Terminated	1. Binds to S1P and inhibits the ‘find me’ and subsequent stages of efferocytosis. 2. Promotes M1 polarization of TAM and remodels the immune microenvironment. 3. Promotes the maturation of DCs, causing them to lose their phagocytosis ability while enhancing their antigen‐presenting function. 4. Reduce tumor volumes, new blood vessel formation, and metastatic potential.	[225, 226]
		Monotherapy	Advanced solid tumors	I	NCT01488513	Completed		
ABC294640	Selective SphK‐2 inhibitor	Monotherapy or combination with hydroxychloroquine sulfate	Advanced cholangiocarcinoma	IIa	NCT03377179	Completed	1. Inhibit SphK2 to reduce SIP production and affect phagocyte migration to apoptotic cells, which reduces the efficiency of efferocytosis. 2. Inhibits the cytoskeletal rearrangement‐related signaling pathway in phagocytes and affects the “eat me” phase of efferocytosis. 3. Inhibit tumor proliferation and migration 4. Promoting autophagy in tumor cells	[227, 228, 266]
		Monotherapy	Relapsed/refractory (R/R) multiple myeloma	Ib/II	NCT02757326	Terminated, with results		
		Monotherapy	Refractory/relapsed MM	Ib/II	NCT02757326	Terminated, with results		

### 2.2 Nucleotides

In the initial phases of apoptosis, Pannexin‐1 (PANX1) is activated through caspase 3/7 cleavage, resulting in the release of ATP and UTP.^[^ ^54^, ^55^ ^]^ This soluble and heat‐resistant “find me” signal acts as a potent inducer, with less than 1% release triggering purinoreceptor‐2 (P2Y2)‐dependent recruitment of phagocytes.^[^ ^56^, ^57^ ^]^ Extracellular nucleotidases convert them into their nonchemotactic diphosphate and monophosphate forms before they can traverse long distances.^[^ ^58^ ^]^ Notably, this process induces the expression of β2 integrin on phagocytes’ surfaces, augmenting the subsequent “eat me” process.^[^ ^59^ ^]^ While it also facilitates the migration of granulocytes (a type of professional phagocyte) to fulfill the “find me” process, the presence of milk fat globule epidermal growth factor‐factor VIII (MFG‐E8), a molecule that links integrin and PS binding, inhibits their migration, dampening their efferocytosis capacity and the accompanying pro‐inflammatory response they orchestrate.^[^ ^60^, ^61^, ^62^ ^]^ Studies highlight that nucleotides released by apoptotic cells can be converted to adenosine by membrane nucleotidases like CD39, CD73, etc., which can then inhibit inflammation by binding to the adenosine receptor.^[^ ^63^ ^]^ Additionally, they can enhance Myc activity via the activation of the DNA‐PKcs‐mTORC2/Rickor pathway, leading to noninflammatory macrophage proliferation and inflammation mitigation.^[^ ^38^ ^]^ Of course, TAMs can release significant amounts of adenosine, upregulate adenosine receptors, triggering a potent anti‐inflammatory response and the secretion of anti‐inflammatory and pro‐inflammatory molecules. As a result, these actions promote M2 polarization and hasten the establishment of an anti‐inflammatory TME.^[^ ^64^ ^]^ However, all these findings suggest that in the early stages of apoptosis, the release of small amounts of nucleotides exerts an anti‐inflammatory effect. If the recognition and phagocytosis of early ACs are hindered, the release of a large amount of ATP (>1 μM) during the late apoptotic process may have a pro‐inflammatory effect.^[^ ^13^ ^]^ Regrettably, the robust phagocytic capabilities of TAMs lead to the early detection, recognition, and elimination of apoptotic cells upon nucleotide release. Notably, the subsequent degradation and breakdown of apoptotic cells can also yield abundant nucleotides, bolstering macrophage cytoskeletal rearrangements to sustain phagocytosis.^[^ ^65^, ^66^ ^]^

### 2.3 LPC

During apoptosis, caspase‐3 cleaves and activates calcium‐independent phospholipase A2 (iPLA2), which hydrolyzes membranous phosphatidylcholine into LPC.^[^ ^39^ ^]^ Disrupted membrane asymmetry also prompts secretory phospholipase A2 (sPLA2) to hydrolyze phosphatidylcholine from the outer membrane leaflet, yielding LPC.^[^ ^39^, ^67^ ^]^ LPC is then released by the ATP‐binding cassette transporter A1 (ABCA1), initiating chemotaxis by binding to G protein‐coupled receptors (GPCRs) G2A and GPR4 on macrophage surfaces.^[^ ^68^, ^69^, ^70^ ^]^ Interestingly, in contrast to other “find me” signals, LPC binds to immunoglobulin (Ig)M antibodies on phagocytes’ surfaces upon complement activation, facilitating the phagocytosis, and clearance of apoptotic cells.^[^ ^71^ ^]^ Thus, LPC may serve a dual role as both a “find me” and “eat me” signal, laying a robust foundation for the subsequent “eat me” process.

### 2.4 CX3CL1

CX3CL1, known as fractalkine, is secreted from ACs through caspase‐ and Bcl2‐regulated mechanisms, serving as a chemokine and intercellular adhesion molecule that binds to the G protein‐coupled receptors CX3CR1. This binding attracts macrophages toward ACs, facilitating the “find me” process.^[^ ^72^ ^]^ Importantly, CX3CL1 secretion also triggers the expression of MFG‐E8 and reinforces integrin binding to ACs.^[^ ^73^ ^]^ Moreover, studies suggest that CX3CL1‐CX3CR1 signaling reduces inflammatory cytokine expression and boosts antioxidant factor production, expediting the establishment of an anti‐inflammatory TME and fostering tumor progression.^[^ ^74^, ^75^, ^76^ ^]^ Consequently, inhibiting this pathway also suppresses efferocytosis. The release of “find me” signals ensures the recognition and localization of ACs by TAMs, prompting the respective receptors on TAM surfaces to interact with ACs.^[^ ^77^, ^78^ ^]^ Besides these defined “find me” signals, Professor Kodi S. Ravichandran's team uncovered that early ACs secrete over 100 soluble metabolites that regulate immune and metabolic gene expression in surrounding cells.^[^ ^79^ ^]^ The existence of additional “find me” or “eat me” signals remains uncertain, necessitating further investigation to address this question.

## 3 “Eat Me” Signals, Which Ensure Specific Recognition of ACs

**Figure 3:**
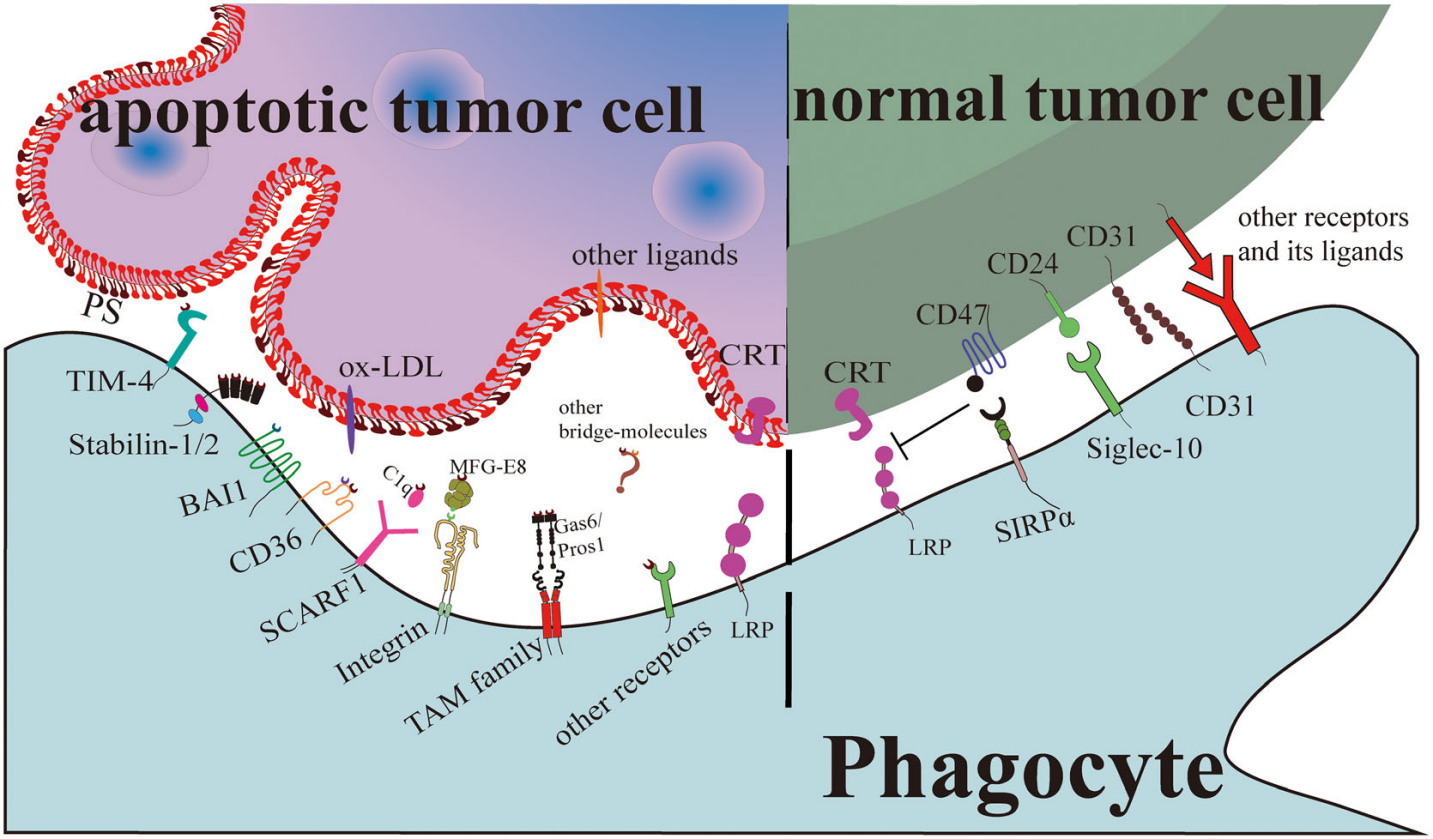
“Eat me” signals ensure specific recognition of ACs. Once phagocytes detect apoptotic cells, the “eat me” signal, typically PS, binds specifically to the corresponding receptor on the phagocyte's surface. This interaction triggers a cytoskeletal reorganization in the phagocyte, facilitating the envelopment of the apoptotic cell in preparation for digestion. In the case of “live” tumor cells, phagocytosis and digestion can be viewed as a potential approach for tumor treatment. Regrettably, surface markers like CD47, CD24, and other “do not eat me” signals on tumor cells bind to their respective receptors on phagocytes, obstructing this process.

TAMs are attracted by the abundance of “find me” signals released by tumor ACs, but the precise recognition of ACs hinges on the “eat me” signals they exhibit. These signals encompass phosphatidylserine (PS), calreticulin (CRT), and oxidized low‐density lipoprotein (ox‐LDL), among others.^[^ ^80^, ^81^ ^]^ Consequently, TAMs’ surface‐specific receptors bind to these signals, initiating subsequent phagocytosis.^[^ ^82^ ^]^ Importantly, this phase of the process also involves bridging molecules like Gas6, Pros1, MFG‐E8, and C1q (**Figure** **3**).^[^ ^83^ ^]^

### 3.1 PS and Its Receptors

Phosphatidylserine (PS) is typically absent from the outer surface of healthy cells, kept on the inner leaflet of the plasma membrane through the controlled actions of specific enzymes known as flippases and scramblases, which rely on regulated and energy‐dependent processes.^[^ ^13^ ^]^ During apoptosis initiation, caspase‐3 activation leads to the inactivation of the flippase adenosine triphosphatase type 11C (ATP11C) and the activation of the scramblase Xk‐related protein 8 (Xkr8), resulting in the irregular exposure of PS on the surface of ACs.^[^ ^84^, ^85^, ^86^ ^]^ Research has shown that PS exposure on ACs can spike to over 240 picomoles/million cells within 2 h after apoptosis induction via anti‐Fas or cephalosporin treatment, compared to less than 0.9 picomoles/million cells on living cells.^[^ ^87^ ^]^ PS exposure stands as the quintessential signal of ACs and remains the most extensively studied “eat me” signal.^[^ ^88^ ^]^ Although PS exposure is also observed in necrotic cells, inducing a pro‐inflammatory immune response upon phagocytosis,^[^ ^89^ ^]^ the robust phagocytic capability of numerous M2‐like TAMs within the TME averts this response by effectively engulfing and digesting ACs before they reach secondary necrosis.

The PS receptors on TAM surfaces primarily interact with PS through two mechanisms: direct and indirect binding. Direct binding involves molecules like TIM‐4, BAI1, and Stabilin that directly bind to PS on AC surfaces, while indirect binding occurs with the aid of soluble bridging molecules to facilitate efferocytosis. TAM family receptors, for instance, collaborate with bridging molecules like Gas6 and Pros1 to establish the connection.^[^ ^80^, ^81^, ^82^, ^83^ ^]^ Notably, these soluble bridging molecules play pivotal and distinct roles in this stage of the process. This is mainly due to: 1) connecting phagocytes to ACs and enhancing the specific recognition of the latter. 2) Activating backbone rearrangement‐related signaling pathways within phagocytes to promote phagosome formation. 3) Acting as an anti‐inflammatory factor itself to regulate the inflammatory response and accelerate the formation of an anti‐inflammatory microenvironment.^[^ ^14^, ^90^, ^91^ ^]^

#### 3.1.1 T‐Cell Immunoglobulin and Mucin (TIM) Receptors

The TIM family comprises immunoregulatory molecules linked to allergic diseases, first detected in mice with a total of eight genes, and just three human TIM genes.^[^ ^92^ ^]^ Since TIM‐1/3 predominantly expresses in T‐cells rather than TAMs and is not involved in efferocytosis, this discussion will not delve into those aspects.^[^ ^93^, ^94^ ^]^ TIM‐4's structure was revealed in 2007 subsequent to its initial identification in the splenic rim and lymphoid organs in 2004.^[^ ^92^, ^95^ ^]^ TIM‐4, a type I transmembrane protein, features a mucin domain, a Cys‐rich immunoglobulin variant (IgV) domain, a transmembrane domain, and an intracellular region. It is predominantly present on the surfaces of macrophages and dendritic cells (DCs) and actively facilitates efferocytosis by directly binding to PS on apoptotic cell surfaces via the CC’FG‐binding cleft in its IgV region, which relies on a metal ion‐dependent ligand‐binding site.^[^ ^94^, ^95^ ^]^ Studies have shown that resident peritoneal macrophages deficient in TIM‐4, along with Kupffer cells (hepatic phagocytes), struggle to engulf apoptotic cells, indicating TIM‐4's indispensable role in efferocytosis.^[^ ^96^ ^]^ Lamentably, owing to the absence of tyrosine kinase phosphorylation sites in TIM‐4's intracellular region, it lacks the capacity for cell signaling and is deemed a tethering receptor in efferocytosis.^[^ ^97^, ^98^ ^]^ Presumably, this unique characteristic underpins its high‐affinity binding to PS. Furthermore, it drives actin cytoskeleton reorganization and the formation of pseudopods facilitating apoptotic cell engulfment via interactions with the integrin^[^ ^99^, ^100^ ^]^ or TAM families^[^ ^101^, ^102^ ^]^ to complete the “eat me” process. Notably, monoclonal antibodies targeting TIM‐4 inhibit the phagocytic activity of TIM‐4^+^ peritoneal macrophages.^[^ ^94^ ^]^ Interestingly, TIM‐4 protein exhibits close associations with human malignancies and is markedly overexpressed in malignant tissues like ovarian cancer,^[^ ^103^ ^]^ glioma,^[^ ^104^ ^]^ and nonsmall cell lung cancer (NSCLC).^[^ ^99^ ^]^ Its involvement in disease recurrence, metastasis, EMT, and its correlation with clinical staging, pathological grading, and cancer prognosis have been well‐documented. Currently, employing an anti‐TIM4 coadministration approach with anticancer vaccines enhances cytotoxic CD8^+^ T‐cell infiltration, contributing to tumor regression and yielding promising results in tumor therapy.^[^ ^105^ ^]^

#### 3.1.2 Stabilin Receptors

Stabilin‐1 and Stabilin‐2 are type I membrane proteins featuring an extracellular region composed of four repeating units, each containing an epidermal growth factor repeat sequence (EGFrps) responsible for binding to PS.^[^ ^106^ ^]^ Stabilin‐1, prevalent in M2‐type macrophages, serves as an M2 marker.^[^ ^107^ ^]^ Upon PS recognition and binding, it triggers its intracellular asparagine‐proline‐x‐phenylalanine (NPxF) motif (where × is any amino acid) to engage the phosphotyrosine‐binding domain of Gulp1, activating Rac1 and facilitating efferocytosis.^[^ ^108^ ^]^ Intriguingly, Stabilin‐1 also possesses an intracellular aspartate‐aspartate serine‐leucine (DDSLL) motif, crucial for mediating efferocytosis upon PS binding.^[^ ^106^ ^]^ Studies revealed that efferocytosis was significantly impeded by anti‐Stabilin‐1 antibodies or short hairpin RNA (shRNA).^[^ ^109^ ^]^ Noteworthily, Stabilin‐1 plays a pivotal role in inflammation and immune response regulation, as knockdown of Stabilin‐1 led to heightened inflammatory cytokine production and prompted T‐lymphocytes to increase interferon (IFN)‐γ levels while reducing interleukin (IL)‐4 and IL‐5 production compared to control cells.^[^ ^110^ ^]^

Stabilin‐2 is present on human monocyte‐derived macrophages (HMDM) surfaces.^[^ ^106^ ^]^ Like Stabilin‐1, Stabilin‐2 can activate its asparagine‐proline‐x‐tyrosine (NPxY) motif (where × is any amino acid) following EGFrps binding to PS, which interacts with Gulp1, thereby activating Rac1.^[^ ^111^, ^112^ ^]^ Additionally, Stabilin‐2 can bind to integrin β5 via its fasciclin 1 (FAS1) domains, culminating in focal adhesion kinase (FAK) activation through GULP‐independent phagocytosis, recruiting a CrkII/ELMO1/DOCK180 complex that activates Rac1, enabling subsequent phagocytosis.^[^ ^112^, ^113^, ^114^ ^]^ Notably, masking Stabilin‐2 with PS‐containing liposomes significantly impaired AC phagocytosis by HMDM or Stabilin‐2‐expressing cells.^[^ ^113^ ^]^ Analogously, knocking down endogenous GULP or introducing a Stabilin‐2 inhibitor yielded comparable effects.^[^ ^115^ ^]^ Remarkably, Stabilin‐2 expression serves as a crucial predictive factor for overall survival (OS) and disease‐free survival in NSCLC patients, while also indicating lymph node metastatic status in solid tumors such as of the tongue, liver, gastric, and colorectal regions.^[^ ^116^, ^117^, ^118^ ^]^ Thus, modulating Stabilin receptor expression represents a viable approach to tumor therapy.

#### 3.1.3 BAI1

Brain angiogenesis inhibitor 1 (BAI1) is a seven‐transmembrane protein classified within the adhesion‐type GPCR family, found to be expressed in primary human monocytes and macrophages.^[^ ^119^ ^]^ BAI1 comprises an extracellular domain, a seven‐transmembrane heptahelical structure, and an intracellular region. Serving as a PS receptor class, BAI1 facilitates PS binding through its thrombospondin type 1 repeats (TSRs) located in the extracellular domain. Diminished BAI1 expression or interference with its function has been shown to impede AC phagocytosis in vivo.^[^ ^119^ ^]^ In murine models of dextran sodium sulfate‐induced colonic inflammation, reduced BAI1 expression correlated with poorer prognoses. Conversely, transgenic mice overexpressing BAI1 exhibited reduced inflammatory markers, decreased AC presence, and disease remission, emphasizing the crucial role of BAI1‐mediated efferocytosis in maintaining homeostasis.^[^ ^120^ ^]^ Given the significance of neovascularization in tumor progression, research demonstrates that heightened BAI1 expression, coinciding with increased TSR levels, can hinder angiogenesis, paradoxically fostering efficient efferocytosis. Conversely, complete BAI1 inhibition can prompt the formation of more aggressive glioblastomas.^[^ ^121^, ^122^, ^123^ ^]^ Thus, further elucidating BAI1's role, exploring the strength of its PS affinity, and assessing the necessity of targeting BAI1 remain paramount considerations for future research endeavors.

#### 3.1.4 CD36

CD36 belongs to the class B scavenger receptor family and exhibits widespread expression throughout the body. It consists of two transmembrane structural domains, a sizable extracellular loop housing a ligand binding site, and two brief cytoplasmic tails at the amino‐ and carboxy‐terminal ends.^[^ ^124^ ^]^ Notably, CD36, functioning as a multiligand scavenger receptor, not only recognizes and binds to PS but also interacts with other “eat me” signals like ox‐LDL exposed on apoptotic cells.^[^ ^125^, ^126^ ^]^ Transcriptional enhancement of various efferocytosis‐associated molecules via the PPAR‐γ signaling pathway sustains efferocytosis.^[^ ^127^ ^]^ In vivo models, such as those inducing lung injury with bleomycin or promoting atherogenesis, underscore the necessity of CD36 for apoptotic cell clearance.^[^ ^124^, ^128^, ^129^ ^]^ Furthermore, CD36 deficiency results in delayed parasitized erythrocyte clearance, intensifying malaria severity.^[^ ^130^ ^]^ Fundamental investigations have revealed that CD36 fosters tumor proliferation, invasion, migration, and stemness by engaging in diverse pathways such as AKT/GSK‐3β/β‐catenin, JAK1/2 phosphorylation, the STAT3/NF‐κB signaling axis, and AMPK modulation.^[^ ^131^, ^132^, ^133^ ^]^ While CD36 is expressed in immune cells like CD8^+^ T‐cells and Treg cells, CD36 on CD8^+^ T‐cell surfaces, recognizing ox‐LDL and engulfing apoptotic cells, heightens intracellular lipid peroxidation, inducing Ferroptosis (a programmed cell death form) detrimental to tumor therapy.^[^ ^134^ ^]^ PS binding triggers the PPAR‐β pathway in tumor‐infiltrating Treg cells, fostering metabolic Treg cell adaptation, inhibiting apoptosis, and establishing an immunosuppressive microenvironment.^[^ ^135^ ^]^ Significantly, as a transporter protein, CD36 facilitates fatty acid (FA) transport to sustain rapid tumor cell growth,^[^ ^127^, ^136^, ^137^ ^]^ culminating in adverse clinical outcomes and exacerbated clinicopathological characteristics.^[^ ^133^, ^138^, ^139^, ^140^ ^]^ In conclusion, the promising strategy of cancer treatment involves targeting CD36.

#### 3.1.5 TAM Receptor Family

The TAM receptor tyrosine kinases (RTKs) family comprises three members: Mertk, Axl, and Tyro3, all membrane receptors with analogous structures.^[^ ^141^ ^]^ Phagocytosis involves PS recognition on apoptotic cell surfaces, facilitated by two bridging molecules, protein S (Pros1) and growth arrest‐specific 6 (Gas6) — both vitamin K‐dependent proteins ubiquitously expressed in the body.^[^ ^142^, ^143^ ^]^ Through γ‐carboxylation of the glutamic acid residues (Gla domain) in their amino‐terminal structure, crucial for Ca^2+^ binding, these molecules enhance PS binding, elevate ligand affinity, and bolster interactions with the TAM receptor through their carboxy‐terminal sex hormone‐binding globulin (SHBG)‐like domains’ laminin G‐like segments, amplifying TAM receptor‐mediated efferocytosis.^[^ ^144^, ^145^ ^]^ Extensive research underscores the TAM receptor and its ligands’ significant role in M2 polarization, pivotal for establishing an immunosuppressive TME.^[^ ^146^, ^147^, ^148^, ^149^ ^]^ This modulation extends to nuclear receptor pathways (PPAR‐γ, PPAR‐δ, RXRα), upregulating Mertk and Axl mRNA levels and enhancing Gas6 secretion,^[^ ^148^, ^150^, ^151^ ^]^ thus perpetuating a self‐sustaining autocrine loop and fostering robust efferocytosis.

Notably, aberrant expression of TAM receptors is quite evident across hematologic and solid tumors and is regarded as a tumor marker.^[^ ^152^, ^153^, ^154^, ^155^ ^]^ Through indirect interactions with peripheral ACs, TAM receptors engage classical oncogenic signaling pathways like MEK/ERK, PI3K/AKT, and JAK/STAT to drive tumorigenesis, cell proliferation, apoptosis resistance, and survival via BCL‐2 and ACK1 pathways.^[^ ^156^, ^157^ ^]^ They also upregulate proteins like RHO, FAK1, and MMP9 to modulate migration and invasion,^[^ ^158^, ^159^, ^160^ ^]^ alongside promoting Snail and Slug expression to induce EMT,^[^ ^158^, ^161^ ^]^ ultimately culminating in unfavorable consequences.^[^ ^155^, ^162^, ^163^, ^164^ ^]^ Then, the question arises as to whether the activation of TAM receptors in phagocytes can achieve similar effects in promoting proliferation, antiapoptosis and accelerate the formation of an immunosuppressive TME under the influence of a large number of anti‐inflammatory factors produced in the subsequent digestion stage. This is an interesting question. Regrettably, the intracellular activation of TAM family receptors within phagocytes has not received much attention. However, for tumor cells, pharmacologically inhibiting Mertk demonstrated inhibition of melanoma growth, invasion, and colony formation in vivo and in vitro.^[^ ^165^ ^]^ Tyro3 knockdown via small interfering RNA (siRNA) impeded breast cancer cell growth, proliferation, and progression through the G0–G1/S cell cycle phase.^[^ ^166^, ^167^ ^]^ Axl inhibition yielded promising results in attenuating tumor cell viability, colony formation, invasion, migration, EMT, and enhancing chemosensitivity.^[^ ^156^, ^158^, ^168^ ^]^ Based on the above studies, we are confident in inhibiting the various pro‐cancer mechanisms (including efferocytosis) involved in the activation of TAM receptors within phagocytes. TAM receptor suppression represents a meaningful approach in novel tumor therapies. Multiple small molecule inhibitors targeting TAM receptors have shown promising preclinical efficacy and progressed through various clinical trial stages (**Table** **2**).

**Table 2:** Targeting the “eat me” phase.

Medicine	Type	Method	Disease	Stage	ClinicalTrials.gov Identifier	Status	Mechanism	Source
Bavituximab	Anti‐PS antibody	Combination with docetaxel	Advanced nonsquamous NSCLC	III	NCT01999673	Completed	1. Inhibits phagocytosis by targeting PS and inhibiting the recognition of ACs by phagocytes. 2. Bind to PS and change the conformation of PS on the membrane surface, preventing phagocytes from forming effective phagocytic cups to wrap ACs for efferocytosis. 3. Promotes M1 polarization and promotes maturation of DCs. 4. Increases CD8+ cytotoxic T‐cell infiltration.	[229, 230, 231, 232]
		Combination with radiation and temozolomide	Newly‐diagnosed Glioblastoma	II	NCT03139916	Completed, with results		
		Combination with docetaxel	Advanced or metastatic breast cancer	II	NCT00669591	Completed		
		Monotherapy	Advanced Solid Tumor	I	NCT00129337	Completed		
		Combination with carboplatin and paclitaxel	Advanced or metastatic breast cancer.	II	NCT00669565	Completed		
		Combination with pembrolizumab	Recurrent/metastatic head and neck squamous cell carcinoma (HNSCC)	II	NCT04150900	Active, not recruiting		
		Combination with pemetrexed and carboplatin	Stage IV NSCLC	Ib	NCT01323062	Completed		
		Combination with pembrolizumab	Advanced HCC	II	NCT03519997	Active, not recruiting		
Bemcentinib (BGB32/R428)	Selective Axl inhibitor	Combination with docetaxel	Advanced NSCLC	I	NCT02922777	Completed	1. Inhibits ALX, which affects the activity of phagocytes themselves and their recognition and phagocytosis of ACs, thereby inhibiting efferocytosis. 2. Inhibits efferocytosis by regulating cytoskeletal rearrangement‐related signaling molecules and disrupting the morphology of phagocytes. 3. Affects cytokines secreted by immune cells and reduces efferocytosis efficiency. 4. Inhibit tumor proliferation, invasion, migration, angiogenesis and EMT. 5. Enhance drug sensitivity.	[234, 235]
		Combination with pembrolizumab	Advanced NSCLC	II	NCT03184571	Completed		
		Monotherapy	AML,high/low‐risk MDS	II	NCT03824080	Completed		
		Combination with pacritinib	Advanced lung adenocarcinoma	Ib/II	NCT06516887	Not yet recruiting		
		Combination with erlotinib	Stage IIIb/IV NSCLC	I/II	NCT02424617	Completed		
		Combination with pembrolizumab	Advanced and unresectable/metastatic triple‐negative breast cancer	II	NCT03184558	Terminated, with results		
		Combination with cytarabine or decitabine	AML or MDS	Ib/II	NCT02488408	Completed, with results		
		Combination with nab‐paclitaxel (GnP)/gemcitabine	Metastatic pancreatic adenocarcinoma.	Ib/II	NCT03649321	Terminated, with results		
		Combination with pembrolizumab/carboplatin/pemetrexed	Untreated advanced/metastatic nonsquamous NSCLC	Ib/II	NCT05469178	Recruiting		
TP‐0903	Axl inhibitor	Monotherapy or combination with ibrutinib	Chronic lymphocytic leukemia small lymphocytic lymphoma	I/II	NCT03572634	Terminated, with results	1. Binding to PS prevents phagocytes from effectively recognizing ACs to inhibit efferocytosis. 2. Blocking phagocytosis by hindering cytoskeletal rearrangement of phagocytes. 3. Interfering with the secretion of cytokines by T‐cells and natural killer cells leads to the decline in the activity of phagocytes and efferocytosis. 4. Inhibit the proliferation and metastasis of tumor cells. 5. Enhance the sensitivity of chemotherapeutic drugs.	[236, 237, 238, 239]
		Monotherapy	Advanced solid tumors, EGFR (+) NSCLC, colorectal carcinoma, recurrent ovarian carcinoma, BRAF‐mutated melanoma	I	NCT02729298	Completed		
		Monotherapy or combination with azacitidine	FLT3‐mutated AML	Ib/II	NCT04518345	Completed		
Cabozantinib	Multireceptor tyrosinase inhibitor	Monotherapy	RET, ROS1, or NTRK fusions; MET or AXL activity advanced NSCLC	II	NCT01639508	Recruiting	1. By mediating the MET pathway to reduce the number of phagocytes themselves. 2. Change the status of the tumor vasculature and affect the migration of phagocytes to ACs, thus hindering efferocytosis. 3. Affecting the signal transduction within phagocytes and thus hindering efferocytosis. 4. Inhibition of cancer cell growth, neovessel formation, and metastasis.	[240, 241]
ONO‐7475	Axl‐Mertk dual‐target inhibitor	Monotherapy or combination with venetoclax	Acute leukemias or MDS	I/II	NCT03176277	Terminated, with results	1. Regulates the secretion of cytokines in the TME and key signaling pathways in phagocytes to reduce efferocytosis efficiency. 2. Inhibits efferocytosis by affecting PS receptors on the surface of phagocytes and typical features of ACs. 3. Reduces cancer cell proliferation 4. Enhance drug sensitivity	[242]
		Monotherapy or combination with ONO‐4538	Advanced or metastatic solid tumors	I	NCT03730337	Completed		
		Osimertinib	EGFR (+) NSCLC	I	NCT06525246	Active, not recruiting		
		Combination with GnP	Pancreatic cancer	I	NCT06532331	Recruiting		
AVB‐S6‐500(Batiraxcept)	AXL inhibitor	Combination with avelumab	Advanced urothelial carcinoma	I	NCT04004442	Active, not recruiting	1. Inhibits efferocytosis by inhibiting ALX‐related signaling pathways and the dynamic balance of Ca^2+^. 2. Affects angiogenesis within the TME, induces hypoxia, and decreases phagocyte skeletal rearrangement and motility. 3. Inhibition of cancer cell invasion and metastasis 4. Enhance drug sensitivity	[243]
		Combination with pegylated liposomal doxorubicin or paclitaxel	Platinum‐resistant Recurrent Ovarian Cancer	I	NCT03639246	Completed		
		Combination with paclitaxel	Recurrent high‐grade uterine cancer	I	NCT05826015	Not yet recruiting		
		Combination with durvalumab	Platinum‐resistant, recurrent epithelial ovarian cancer	I/II	NCT04019288	Active, not recruiting		
		Combination with paclitaxel	Platinum‐resistant ovarian cancer	III	NCT04729608	Terminated		
MRX‐2843	MERTK/ FLT3 inhibitor	Monotherapy	R/R AML	I/II	NCT04946890	Not yet recruiting	1. Regulate the function of efferocytosis receptor on the surface of phagocytes and the transduction of intracellular signaling pathway, which affects phagocyte skeleton rearrangement and the formation of LAP and prevents the endocytosis and digestion of ACs. 2. Regulate the oxygen content in TME and cytokines secreted by immune cells to affect efferocytosis. 3. Inhibits colony formation, reduce tumor size 4. Increase drug sensitivity 5. Prolonged survival	[244]
		Monotherapy	R/R advanced and/or metastatic solid tumors	I	NCT03510104	Active, not recruiting		
		Combination with osimertinib	Advanced EGFR mutant NSCLC	I	NCT04762199	Recruiting		
PF‐07 265 807	selective inhibitor of Mertk and Axl	Monotherapy or combination with sasanlimab and/or axitinib	Advanced metastatic solid tumors	I	NCT04458259	Active, not recruiting	1. Regulates PS receptor function on the surface of phagocytes and intracellular signaling pathway transduction to affect efferocytosis. 2. Enhancing the effectiveness of immunotherapy.	[245]

#### 3.1.6 Integrin Receptors

The integrin family comprises heterodimers, each composed of two α and β chains (or subunits) linked by noncovalent bonds. MFG‐E8, also known as lactoferrin, is a pleiotropic glycoprotein weighing around 75–80 kDa with anti‐inflammatory attributes. It encompasses two epidermal growth factor (EGF)‐like domains, a mucin‐like domain, and two factor‐VIII‐homologous domains (C1 and C2).^[^ ^25^, ^61^, ^169^, ^170^ ^]^ The second EGF domain features an arginine–glycine–aspartate (RGD) motif that binds to αvβ3 and αvβ5 integrins. The C‐domains serve as PS‐binding aminophospholipid sites.^[^ ^169^ ^]^ Beyond assisting Tim‐4 in executing efferocytosis, αvβ3 and αvβ5 integrins on phagocyte surfaces can directly interact with PS on apoptotic cell surfaces via MFG‐E8, thereby facilitating AC‐dependent uptake.^[^ ^100^, ^171^, ^172^ ^]^ Additionally, MFG‐E8 aids in activating the SH2 domains of integrin‐associated Src family kinases post PS attachment, subsequently regulating pro‐/anti‐inflammatory mediator production through multiple pathways governed by FAK phosphorylation at Tyr397.^[^ ^169^, ^173^, ^174^ ^]^ MFG‐E8 can originate from various sources, such as CX3Cl1 “find me” signaling released by apoptotic cells, contents postapoptotic cell digestion, and TAM itself, all capable of upregulating MFG‐E8 expression.^[^ ^61^, ^73^, ^171^, ^175^ ^]^ Several studies have illustrated the critical role of MFG‐E8 in αvβ3 or αvβ5 integrin‐mediated efferocytosis.^[^ ^175^, ^176^, ^177^ ^]^ Notably, MFG‐E8 also functions as a growth factor involved in angiogenesis and immune tolerance.^[^ ^178^ ^]^ Consequently, inhibiting MFG‐E8, αvβ3 or αvβ5 expression could hold therapeutic promise in tumor treatment. Research indicates that extracellular divalent cations (e.g., Ca^2+^, Mg^2+^) modulate integrin function by influencing its conformation. Treatment of wild‐type bone marrow‐derived macrophages with ethylenediaminetetraacetic acid (a divalent cation chelator) resulted in over 90% reduction in PS‐containing particle uptake.^[^ ^100^ ^]^ RGD‐containing peptides competitively hinder ligand binding to integrins, impeding regular efferocytosis. Notably, mutation of the RGD motif in MFG‐E8 and silencing of αvβ3 or αvβ5 also disrupt normal efferocytosis.^[^ ^172^ ^]^ While relevant drug development has not reached preclinical trials, these findings offer a foundation for potential future drug discovery initiatives.

#### 3.1.7 Scavenger Receptor Class F Member 1 (SCARF1)

SLE is characterized by impaired clearance of ACs and immune complexes in vivo, with a pivotal role attributed to component C1q.^[^ ^179^ ^]^ C1q acts as a bridging molecule essential for the recognition and binding of PS by the phagocyte surface SCARF1.^[^ ^179^, ^180^ ^]^ However, in some SLE patients, the presence of autoantibodies against SCARF1 hampers efficient efferocytosis, diminishes DNASE1L3 activity, and exacerbates SLE progression.^[^ ^181^ ^]^ This scenario prompts consideration of isolating anti‐SCARF antibodies from such patients for potential cancer treatment, emphasizing the necessity for further research into this efferocytosis pathway.

In addition to the noted PS receptors, emerging players such as triggering receptor expressed on myeloid cells‐like protein 2 (TLT2) and the receptor for advanced glycation end‐products (RAGE) also interact with PS to facilitate the “eat me” process.^[^ ^182^, ^183^ ^]^ Understanding their roles in depth holds promise for devising novel tumor treatment strategies.

### 3.2 Calreticulin and Its Receptors

Normal cells evade phagocytosis, whereas tumor cells engage in this process primarily due to the presence of calreticulin (CRT) on their surface—an essential “eat me” signal.^[^ ^184^, ^185^ ^]^ Conversely, the presence of CD47, acting as a “do not eat me” signal on tumor cells, inhibits phagocytosis.^[^ ^186^ ^]^ Research indicates promising outcomes in tumor treatment using anti‐CD47 monoclonal antibodies, as they enhance phagocytosis of nonapoptotic tumor cells by promoting subsequent interactions between CRT and low‐density lipoprotein receptor‐related protein (LPR).^[^ ^187^, ^188^ ^]^ Although heightened CRT expression on cancer cell surfaces during apoptosis intensifies efferocytosis, prompt phagocytosis of apoptotic cells accelerates the creation of an immunosuppressive TME.^[^ ^189^, ^190^ ^]^ Therefore, a tailored approach balancing phagocytosis and nonphagocytosis of tumor cells based on their status holds potential for optimizing tumor treatment outcomes. CRT, akin to other key efferocytosis receptor molecules, is notably expressed on the surfaces of neuroblastoma, bladder cancer, non‐Hodgkin's lymphoma, various hematologic malignancies, and solid tumors. Elevated levels of CRT mRNA in different tumor types correlate with inferior clinical prognoses.^[^ ^186^ ^]^ Mechanistically, CRT triggers the PI3K and ERK pathways with the assistance of Galphai2 proteins, promoting tumor cell proliferation and survival.^[^ ^185^ ^]^ The tumorigenic impact of CRT is further linked to diminished cell adhesion and immune evasion through compromised MHC class I antigen presentation.^[^ ^191^, ^192^ ^]^ Overall, selective phagocytosis of nonapoptotic tumor cells proves advantageous for tumor therapy, while phagocytosis of apoptotic cells yields contrasting effects.

### 3.3 Other “Eat Me” Signals and Their Receptors

Intercellular adhesion molecule 1 (ICAM1), abundantly expressed in colonic mucosal macrophages from colitis mice and IBD patients, plays a crucial role in promoting efferocytosis and maintaining homeostasis. Notably, ICAM1‐deficient mice and ICAM1‐knockdown macrophages demonstrate significantly impaired efferocytosis, highlighting the significance of ICAM1 in this process.^[^ ^193^ ^]^ Additional “eat‐me” signals, including annexin 1, thrombospondin 1 binding sites, and complement proteins C3b, actively participate in efferocytosis as well.^[^ ^13^, ^194^, ^195^ ^]^ Nonetheless, our current understanding of these signals remains incomplete. Just as human heights and weights vary, the exposure of “eat‐me” signals on ACs and efferocytosis receptors on TAMs also varies.^[^ ^13^, ^78^, ^194^, ^196^ ^]^ Furthermore, the affinity between distinct PS receptors and their ligands on TAM surfaces differs—such as the strong binding affinity of TIM‐4 for PS compared to the weaker affinities of Stabilin receptor and MFG‐E8.^[^ ^13^ ^]^ Despite these differences, the ultimate goal remains consistent: efficient recognition and phagocytosis of ACs to ensure effective efferocytosis. Understanding this diversity underscores the potential for targeting various stages of efferocytosis through multiple approaches.

### 3.4 The “Do Not Eat Me” Signal, Which Protects “Live” ACs

Cancer cells are “sophisticated” in their survival strategies. To evade phagocytosis, much like normal cells, they can express multiple “do not eat me” signals such as CD47, CD31, and CD24. Moreover, they activate multiple downstream signaling pathways, thereby facilitating tumor progression.^[^ ^187^, ^197^, ^198^, ^199^ ^]^ Multiple studies have shown that antagonizing these signals to enhance their phagocytosis has shown remarkable effects in promoting tumor regression.^[^ ^187^, ^188^, ^199^ ^]^ Although phagocytosing non‐ACs violates the original definition of efferocytosis, everything becomes reasonable if we consider them as “living ACs”.

#### 3.4.1 CD47

CD47 is a pentameric transmembrane glycoprotein expressed on all normal cells,^[^ ^187^, ^197^ ^]^ yet it exhibits significantly elevated levels in various cancers, such as myeloid leukemia, bladder cancers, neuroblastoma, non‐Hodgkin's lymphoma, and ovarian cancer, correlating with poor prognosis.^[^ ^200^, ^201^, ^202^ ^]^ Functioning as a “do not eat me” signal, CD47 engages with signaling regulatory protein α (SIRPα) to phosphorylate tyrosine kinase within the phagocyte's cytoplasmic domain. This interaction further recruits and activates the phosphatase SHP1/2, ultimately impeding phagocytosis by inhibiting nonmuscle myosin IIA.^[^ ^203^, ^204^, ^205^ ^]^ Additionally, CD47 activation suppresses inflammatory gene expression, fostering an anti‐inflammatory microenvironment that boosts the expression of other PS receptors, enhancing efferocytosis efficiency and potentially facilitating tumor immune evasion.^[^ ^204^, ^205^, ^206^ ^]^ The use of anti‐CD47 monoclonal antibodies promotes CRT and LPR interaction, reinstating normal phagocytosis and stimulating CD8^+^ T‐cells to exert cytotoxic effects against tumor cells. This mechanism has shown promise in both preclinical models and clinical applications.^[^ ^187^, ^188^, ^207^, ^208^ ^]^ However, caution is advised as CD47 is also present in erythrocytes and normal cells in vivo, necessitating consideration of potential off‐target effects when utilizing CD47 antibodies.^[^ ^205^ ^]^ Despite this, the utilization of anti‐CD47 monoclonal antibodies in tumor treatment is yielding encouraging outcomes.^[^ ^188^, ^207^ ^]^

#### 3.4.2 Other “Do Not Eat Me” Signal

CD24 exhibits high expression on the surface of tumors, hindering phagocytosis by binding to the sialic acid‐binding immunoglobulin‐like lectin 10 (Siglec10) of TAMs.^[^ ^199^ ^]^ CD31 achieves a similar effect through homologous interactions.^[^ ^198^ ^]^ Moreover, various other “do not eat me” signals, such as CD46 and integrin‐associated protein (IAP), are also detected on tumor cell surfaces.^[^ ^194^ ^]^ Nonetheless, the underlying molecular mechanisms remain partially understood, impeding the development of drugs targeting these signals, albeit offering a promising avenue for future research and therapeutic advancements.

## 4 Digestive Phase, Providing Material for Ongoing Efferocytosis

**Figure 4:**
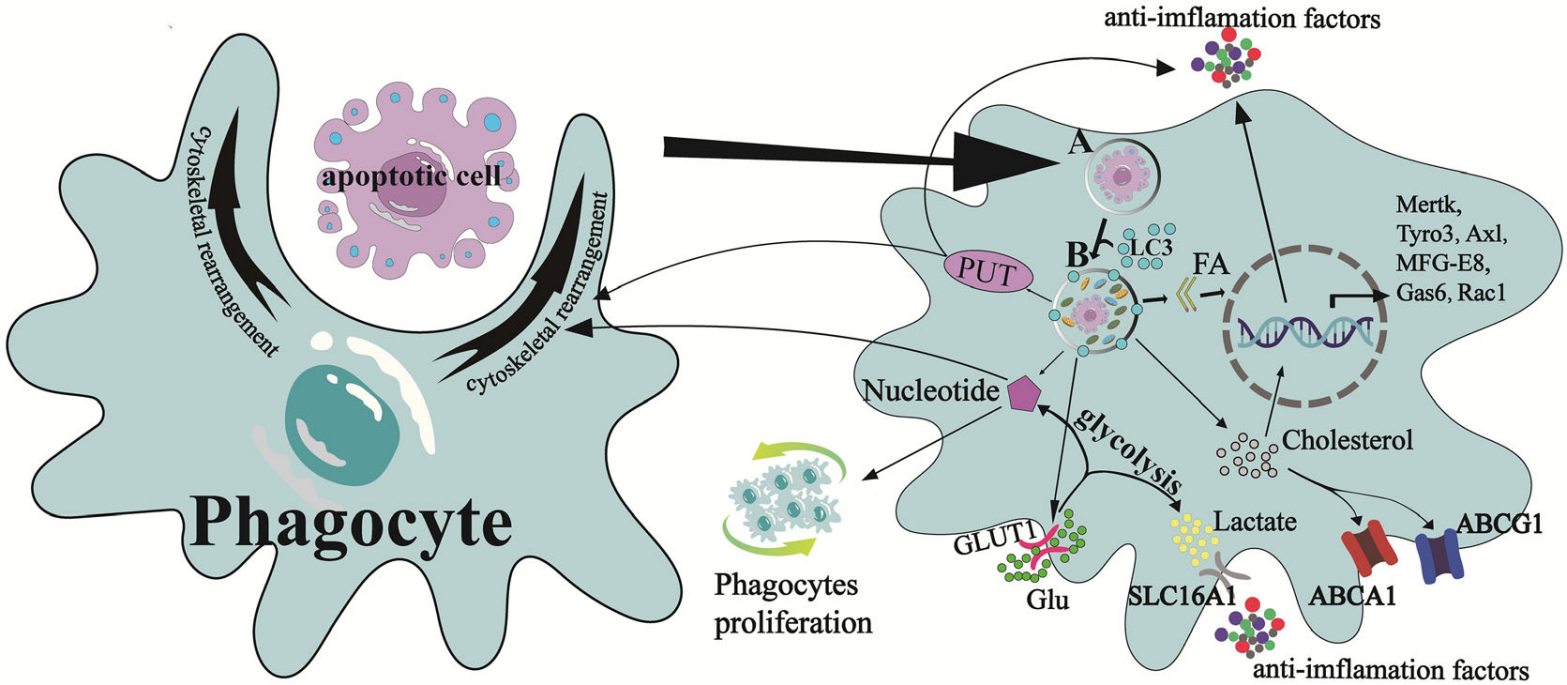
Digestive phase provides material for ongoing efferocytosis. During the digestive phase, which supplies material for continuous efferocytosis, apoptotic cells are discovered, recognized, and engulfed by phagocytes. Subsequently, the resulting phagosomes (A) form LAPs (B) with the assistance of LC3, and the cell contents are digested and broken down by various enzymes. This process regulates the synthesis of inflammatory factors, fosters phagocytosis and M2‐type polarization, upregulates the transcription of efferocytosis‐associated receptors, and replenishes depleted surface materials on phagocytes. Continuous glucose metabolism also ensures a steady energy supply for sustained efferocytosis.

Cell digestion is a complex process involving phagocytic cytoskeletal rearrangement, phago‐lysosomal maturation, and degradation of ACs. Concretely, upon specific recognition of ACs by the “eat me” signaling receptor on the surface of TAMs, Rac‐mediated signaling pathways are activated. This activation leads to the invagination or localized exocytosis of the phagocyte plasma membrane, forming a “phagocytic cup” that engulfs the ACs.^[^ ^80^, ^209^, ^210^, ^211^ ^]^ Subsequently, microtubule‐associated protein 1A/1B light chain 3 (LC3) is recruited to the phagosome surface, assembling to create a LC3‐associated phagocytosis (LAP) body.^[^ ^212^ ^]^ The phagosome then fuses with one or more lysosomes to generate a phago‐lysosome, where the intracellular components undergo degradation through various enzymes such as proteases, nucleases, and lipases. Notably, the involvement of LC3 in this process is crucial for efferocytosis as it accelerates the maturation of the phagocytic polyploid and facilitates the efficient degradation of ACs^[^ ^212^ ^]^ (**Figure** **4**).

Arginine and ornithine can be converted into PUT by intracellular ornithine decarboxylase and arginase in macrophages. This conversion increases the activity of Rac1, promoting macrophage actin nucleation and cytoskeletal rearrangement, or facilitating anti‐inflammatory effects by further transformation into spermidine.^[^ ^24^, ^79^, ^209^, ^213^ ^]^ Additionally, fatty acids (FA) produced can induce the transcription of anti‐inflammatory factors by altering the NAD^+^/NADH balance, thereby activating the sirtuin 1 signaling pathway.^[^ ^13^ ^]^ Ornithine decarboxylase activity can be elevated to boost PUT production.^[^ ^214^ ^]^ The mononucleotide produced during digestion triggers TAM proliferation via the mTORC2/Akt/Myc signaling pathway, ensuring TAM abundance and enhancing efferocytosis by polarizing toward the M2 type in response to various anti‐inflammatory stimuli.^[^ ^38^ ^]^ Moreover, the digestion process upregulates and activates glucose transporter protein (GLUT)‐1, also known as SLC2A1, facilitating the influx of extracellular glucose. This glucose metabolism releases ATP through glycolysis, promoting actin polymerization for sustained efferocytosis, and generates lactate, releasing anti‐inflammatory signals via SLC16A.^[^ ^65^, ^66^, ^215^ ^]^ Cholesterol produced is effluxed to apolipoprotein A‐1 (ApoA‐1) through ABCA1 and ABCG1, with the interaction between ABCA1 and ApoA‐1 demonstrating anti‐inflammatory effects.^[^ ^15^ ^]^ Furthermore, cholesterol can be esterified and hydrolyzed by acidic lipase to form 25/27‐hydroxysterols, modulating the PPARs and liver X receptors (LXRs) pathway to regulate inflammatory factor secretion, along with continuous ABCA1 activation.^[^ ^216^, ^217^, ^218^, ^219^ ^]^ Importantly, this process upregulates the transcription of efferocytosis‐associated genes such as Mertk, Tyro3, Axl, MFG‐E8, Gas‐6, and Rac.^[^ ^38^, ^127^ ^]^ Digestion also replenishes surface substances depleted by phagocytes, ensuring the seamless continuation of efferocytosis.

## 5 Targeting Efferocytosis to Promote Tumor Regression

The TME facilitates the interaction of ligands and receptors during efferocytosis. Thanks to this advantageous setting and the incremental understanding of the efferocytosis process, it has been discovered that inhibiting efferocytosis and promoting secondary necrosis of apoptotic cells in the anti‐tumor immune microenvironment can greatly benefit tumor treatment. Various antibodies and small molecule inhibitors targeting different stages of efferocytosis have been developed, showing promising results in preclinical models and clinical trials.

### 5.1 Targeting the “Find Me” Phase

In the “find me” signal stage, current research primarily centers around S1P. Several tumor treatment drugs have been developed to either reduce its expression or antagonize its receptors, undergoing active preclinical investigations. Notably, drugs like Suramin, Fingolimod (FTY720), and Sonepcizumab (LT1009) have progressed to various stages of clinical research (Table 1). Suramin, a S1P receptor antagonist, was first trialed in the 1980s on patients with advanced‐stage adrenal and renal cancers.^[^ ^220^ ^]^ It exerts antitumor effects by impeding cancer cell activity, tumor invasion, metastasis, angiogenesis, and fibroblast growth factor actions.^[^ ^221^ ^]^ A phase I/II clinical trial (NCT00054028) on stage III or IV metastatic breast cancer patients revealed enhanced antitumor effects when noncytotoxic doses of Suramin were combined with paclitaxel. Encouraging outcomes were also observed in individuals with hormone‐refractory prostate cancer.^[^ ^222^ ^]^ Fingolimod, a S1P receptor modulator, has been extensively studied for treating patients with relapsing forms of multiple sclerosis (MS). It inhibits tumor growth by suppressing SphK1 activity, binding to S1P receptors, thus hindering tumor vasculature, enhancing reactive oxygen species (ROS) production, and elevating T‐lymphocyte levels in the body.^[^ ^223^, ^224^ ^]^ Sonepcizumab, a monoclonal antibody against S1P, has demonstrated efficacy in curtailing tumor growth, invasion, and angiogenesis in various murine models of human cancer. Although its clinical trial in patients with solid tumors (NCT00661414) did not meet the primary endpoint of progression‐free survival (PFS) and led to peripheral blood lymphocytopenia.^[^ ^225^, ^226^ ^]^ ABC294640, a selective SphK‐2 inhibitor, has exhibited significant inhibition of tumor growth in preclinical mouse studies and is presently under investigation for hepatocellular carcinoma (HCC), diffuse large B‐cell lymphoma (DLBCL), Kaposi sarcoma, and other malignancies.^[^ ^227^, ^228^ ^]^

### 5.2 Targeting the “Eat Me” Phase

#### 5.2.1 Targeting PS

Due to ACs characteristically exposing PS and the variety of PS receptors present, targeting PS has emerged as a compelling focus of research interest (Table 2). In preclinical investigations, PS‐targeting agents have exhibited the ability to extend survival by inhibiting tumor growth and enhancing the effectiveness of chemotherapy and radiotherapy across various cancer types.^[^ ^229^, ^230^, ^231^ ^]^ Bavituximab, an anti‐PS antibody, has demonstrated improved survival outcomes in advanced NSCLC by modulating multiple mechanisms. These mechanisms include promoting M1 polarization, increasing secretion of pro‐inflammatory cytokines, fostering dendritic cell maturation, and enhancing tumor‐specific cytotoxic T‐lymphocyte‐mediated immune responses.^[^ ^232^ ^]^ Unlike PS‐binding agents (e.g., annexin V), Bavituximab predominantly binds to PS through the serum cofactor beta‐2 glycoprotein (β2GP1). A recent phase III clinical trial revealed a promising trend with Bavituximab showing potential benefits in terms of overall survival in a subset of patients with pretreatment serum β2GP1 levels ≥200 μg mL^−1^ who subsequently received immune checkpoint inhibitors (ICIs). These results suggest that Bavituximab warrants further exploration due to its potential to enhance outcomes when used in conjunction with ICIs.^[^ ^233^ ^]^

#### 5.2.2 Targeting PS Receptors

Regarding this subsection, much of the ongoing research places emphasis on the TAM family of receptors. Bemcentinib (also known as BGB324 or R428) is a selective Axl inhibitor created by BerGenBio. It operates by impeding tumor metastasis and angiogenesis through the inhibition of Axl and its subsequent Akt phosphorylation.^[^ ^234^ ^]^ Moreover, Bemcentinib has the capability to enhance sensitivity to mitotic inhibitors like Docetaxel, resulting in superior antitumor effects.^[^ ^235^ ^]^ To date, Bemcentinib has exhibited promising efficacy in a diverse array of solid tumors and hematological malignancies, either independently or in combinatorial treatments with other medications (NCT02922777/NCT03184571/NCT02488408). Notably, a Phase II trial (NCT03184571) carried out on platinum‐resistant patients with advanced lung adenocarcinoma showcased a substantial clinical advantage in PFS, with a median PFS of 8.4 versus 2.9 months in patients displaying positive cAXL (cell surface AXL immunohistochemistry composite score). TP‐0903, a novel oral Axl inhibitor, has demonstrated efficacy in restraining tumor cell proliferation, metastasis, and enhancing sensitivity to chemotherapeutic agents across various solid tumors and hematological malignancies.^[^ ^236^, ^237^, ^238^, ^239^ ^]^ Cabozantinib, a multireceptor tyrosine kinase inhibitor, has shown promise in preclinical trials by modulating multiple signaling pathways to impede cancer cell growth, metastasis, and neovascularization. It has exhibited reductions in tumor size in preclinical studies involving medullary thyroid and prostate cancers.^[^ ^240^, ^241^ ^]^ Presently, a phase II clinical trial (NCT01639508) is ongoing in advanced NSCLC patients with various gene fusions or increased MET or AXL activity. ONO‐7475, an Axl‐Mertk dual‐target inhibitor, has demonstrated the capability to overcome Venetoclax resistance in FLT3‐ITD mutant acute myeloid leukemia (AML).^[^ ^242^ ^]^ Additionally, drugs like AVB‐S6‐500 (Batiraxcept),^[^ ^243^ ^]^ MRX‐2843,^[^ ^244^ ^]^ PF‐07265807,^[^ ^245^ ^]^ and others targeting the “eat me” signal have displayed favorable efficacy in treating different types of cancers. Results from these preclinical investigations and clinical trials indicate that combination therapies hold significant promise for the future. Excitingly, antibody‐drug conjugate (ADC) medications like BA3011 and chimeric antigen receptor T‐cell (CAR‐T) therapies like CCT301‐38 have also been developed and are presently undergoing safety and efficacy evaluations.

#### 5.2.3 Targeting the “Do Not Eat Me” Signal

The signal of “don't eat me” on the surface of tumor cells can inhibit phagocytes from digesting them. Blocking these signals and exposing the combination of CRT and LPR can promote the phagocytosis of tumor cells, which is one of the novel anti‐cancer ways. Consequently, several drugs (**Table** **3**) have been developed and are currently undergoing active clinical trials. Magrolimab (Hu5F9‐G4) is a humanized IgG4 antibody that exhibits a high affinity for CD47 and has demonstrated effectiveness in treating AML when combined with Cytarabine, as evidenced in a study (NCT04313881). While it may lead to anemic side effects, the drug can restore hematopoiesis stimulation. Notably, owing to its promising clinical trial outcomes, it has gained FDA approval as a therapeutic option for myelodysplastic syndrome (MDS).^[^ ^246^ ^]^ Ligufalimab (AK117) is a novel CD47 antibody that hinders the growth of blood and solid tumor cell lines. Initial findings suggest that it induces minimal red blood cell hemagglutination compared to Magrolimab.^[^ ^247^ ^]^ Evorpacept (ALX148) serves as a CD47 antagonist, formed through the fusion of a modified SIRPαD1 structural domain with an inactive human IgG1‐Fc. Preclinical studies have illustrated its high‐affinity binding to CD47, enhancing macrophage‐mediated phagocytosis of tumor cells. Moreover, it has the capacity to boost innate and adaptive immunity by facilitating dendritic cell maturation, M1‐type polarization of TAMs, intensifying T‐cell effector function, promoting inflammatory factor production, and augmenting the antitumor efficacy of immunotherapy.^[^ ^248^ ^]^ Based on this, several clinical trials are currently enrolling diverse patient populations, with promising results anticipated. Furthermore, CD47‐targeting antibodies like AO‐176,^[^ ^249^ ^]^ Lemzoparlimab (TJ011133, TJC4),^[^ ^250^, ^251^ ^]^ and HX009^[^ ^252^, ^253^ ^]^ have also progressed to clinical trials, showcasing the expanding landscape of therapeutic options in this domain. As we listed in Table 3, at present, the research on “do not eat me” signal is mostly limited to targeting CD47, but other signal inhibitors such as CD24‐Fc, IMM47, and ATG‐031 have shown promising results in preclinical experiments.^[^ ^254^, ^255^ ^]^ We are looking forward to their final launch, which will bring new hope to cancer patients.

**Table 3:** Targeting the “do not eat me” signal.

Medicine	Type	Method	Disease	Stage	ClinicalTrials.gov Identifier	Status	Mechanism	Source
Magrolimab (Hu5F9‐G4)	Anti‐CD47 antibody	Combination with chemotherapy	Advanced urothelial carcinoma	I	NCT05738161	Recruiting	1. Block the CD47 ‐ SIRPα signaling pathway and increase the activity of phagocytes to enhance phagocytosis of tumor cells. 2. Activate and alter cytokines secreted by T‐cells to enhance phagocytosis of tumor cells. 3. Promote tumor regression. 4. Interfere with the ability of cancer cells to grow and spread.	[246]
		Combination with azacitidine	High‐risk MDS	III	NCT04313881	Terminated		
		Combination with atezolizumab	R/R AML	Ib	NCT03922477	Terminated		
		Combination with avelumab	Solid tumor	Ib	NCT03558139	Completed		
		Combination with other anticancer drugs	R/R MM	II	NCT04892446	Completed		
		Combination with cetuximab	Solid tumors and advanced colorectal cancer	Ib/II	NCT02953782	Completed, Has results		
AK117	Anti‐CD47 antibody	Monotherapy	R/R advanced /metastatic solid tumors or lymphomas	I	NCT04728334	Completed	1. Inhibit the expression of CD47 on the surface of tumor cells and bind to it to promote its internalization and digestion by phagocytes. 2. Regulate the cytokine secretion of T‐cells to enhance the efferocytosis ability. 3. Promote the infiltration of immune cells and inhibit the growth of cancer cells 4. Enhanced drug sensitivity	[247]
		Monotherapy or combination with AK129	R/R classic Hodgkin lymphoma with PD‐1/L1 inhibitor treatment failure	I/II	NCT06642792	Not yet recruiting		
		Combination with azacitidine	MDS	I/II	NCT04900350	Recruiting		
		Combination with azacitidine	Higher‐risk MDS	II	NCT06196203	Recruiting		
		Combination with anti‐EGFR	Recurrent or metastatic HNSCC	II	NCT06508606	Not yet recruiting		
		Combination with azacitidine	AML	Ib/II	NCT04980885	Recruiting		
Evorpacept (ALX148)	CD47 Antagonist	Combination with rituximab and lenalidomide	Indolent and aggressive B‐cell non‐Hodgkin lymphoma	I/II	NCT05025800	Recruiting	1. Enhances phagocytosis of tumor cells by binding to CD47 and altering the signaling pathway mediated by the complement system and the extracellular vesicles released by tumor cells. 2. Promotes the formation of LAP and increases the energy for phagocytosis to accelerate the digestion of tumor cells. 3. Interfering with the growth and spreading ability of cancer cells and enhancing the function of T‐cells. 4. Enhance sensitivity to drugs.	[248]
		Combination with enfortumab vedotin and/or other anticancer therapies	Urothelial carcinoma	I	NCT05524545	Recruiting		
		Combination with pembrolizumab	Advanced HNSCC	II	NCT04675294	Recruiting		
		Combination with liposomal doxorubicin and pembrolizumab	Recurrent platinum‐resistant ovarian cancer	II	NCT05467670	Recruiting		
		Combination with pembrolizumab	Human papilloma virus oropharynx cancer	II	NCT05787639	Recruiting		
		Combination with zanidatamab	Advanced HER2‐expressing cancer	Ib/2	NCT05027139	Active, not recruiting		
		Combination with trastuzumab, ramucirumab, and paclitaxel	Advanced HER2+ gastric cancer	II/III	NCT05002127	Recruiting		
AO‐176	Anti‐CD47 mAb	Monotherapy or combination with Bortezomib/Dexamethasone	R/R MM	I/II	NCT04445701	Completed	1. Enhances CRT expression while blocking CD47, and provides energy to phagocytes for phagocytosis and digestion of tumor cells. 2. Provide materials for phagocyte synthesis by activating the pentose phosphate pathway. 3. Promote the migration of phagocytes to the tumor site and the formation of LAP, which accelerates the metabolism of tumor cells. 4. Directly kill tumor cells 5. Enhance drug sensitivity 6. Increase immune cell infiltration and pro‐inflammatory factor levels	[249]
		Monotherapy or combination with paclitaxel or pembrolizumab	Advanced solid tumors	I/II	NCT03834948	Completed		
Lemzoparlimab (TJ011133, TJC4)	Anti‐CD47 antibody	Monotherapy or combination with azacitidine	AML or MDS	I/IIa	NCT04202003	Completed	1. Binds to CD47 and upregulates CRT expression to promote endocytosis and metabolism of tumor cells. 2. Binds to Fc receptors on the surface of phagocytes and enhances their clearance of the tumor cell. 3. Promote the recruitment and infiltration of immune cells in TME to facilitate the killing of tumor cells. 4. Improve drug sensitivity	[250, 251]
		Monotherapy or combination with pembrolizumab or Rituximab	R/R advanced solid tumors and lymphoma	I	NCT03934814	Completed		
		Combination with azacitidine	High‐risk MDS	III	NCT05709093	Recruiting		
HX009	Anti‐CD47/PD‐1 Bifunctional Antibody	Monotherapy	Advanced solid tumors	1	NCT04097769	Completed Active, not recruiting Not yet recruiting Recruiting	1. Block CD47‐SIRPα and activate related signaling pathways in phagocytes to enhance phagocytosis and degradation of tumor cells. 2. Altering the surface properties of tumor cells to make them more susceptible to phagocytosis. 3. Block the binding of PD‐1 and PD‐L1 to enhance the immunotherapy effect. 4. Increase antigen presentation by tumor cells and inhibition of tumor growth	[252, 253]
		Monotherapy	Advanced solid tumors	I	NCT05731752			
		Combination with IN10018	Advanced solid tumors	I/II	NCT06708663			
		Monotherapy	R/R lymphoma	I/II	NCT05189093			

### 5.3 Other Means for Targeting Efferocytosis

In addition to the phase‐targeted drugs highlighted earlier, the solute carrier (SLC) family represents the second‐largest group of proteins in the human genome and exhibits a high level of druggability.^[^ ^256^ ^]^ Within the realm of efferocytosis, the “find me” signal released by the SLC family serves as an initial marker for this process. Notably, during the efferocytosis process, SLC2A1 facilitates extracellular glucose transport and subsequent gluconeogenesis, SLC16A1 releases lactate produced from glycolysis, and SLC12A2 regulates chloride efflux from metabolites. Collectively, these actions contribute to the perpetuation of efferocytosis.^[^ ^257^ ^]^ Inhibition of the SLC family can impede efferocytosis and potentially play a role in tumor regression, making it an intriguing avenue for exploration.^[^ ^258^ ^]^ The ABC family of transporter proteins is another pivotal component of efferocytosis,^[^ ^15^ ^]^ modulating the exposure of “find me” and “eat me” signals to facilitate efferocytosis. Furthermore, the interaction between ABCA1 and apoA‐I expedites the formation of an immunosuppressive TME.^[^ ^15^ ^]^ Therefore, investigating the inhibition of ABCA1 expression presents a promising area of study. The formation of LAP is crucial for the breakdown and digestion of ACs, hastening the processing of ACs and leading to the subsequent generation of anti‐inflammatory cytokines. This process establishes immune tolerance by inhibiting T‐lymphocyte function.^[^ ^259^, ^260^ ^]^ Disruption of LAP can impede phagosome maturation, triggering the activation of inflammatory signaling pathways and the production of proinflammatory mediators like IL‐1β and IL‐6. Thus, targeting LAP‐specific proteins emerges as a potential therapeutic strategy for combating tumors.^[^ ^212^, ^260^ ^]^ Efforts to induce nonapoptotic and immunogenic cell death in tumor cells, such as through agents like bleomycin, oxaliplatin, cyclophosphamide, anthracyclines, and glycosides, can be beneficial. However, the diverse nature of tumors and the complexity of the human body render these approaches insufficient.^[^ ^261^ ^]^ Other drugs like Thymosin α‐1 (Tα‐1) have the capacity to inhibit efferocytosis by reversing M2 polarization while also enhancing the antitumor effects of chemotherapeutic agents.^[^ ^262^ ^]^ This suggests their potential as adjunctive agents to inhibit efferocytosis. It is anticipated that these interventions will yield positive outcomes in clinical settings.

## 6 Conclusion and Future Perspective

In today's landscape, the primary treatment approach for most cancers continues to revolve around chemoradiotherapy, with surgical resection being a viable option for early‐stage solid tumors. However, this approach often results in increased ACs, thereby exacerbating efferocytosis within the TME and hastening the development of an anti‐inflammatory immunosuppressive TME.^[^ ^25^, ^26^ ^]^ Nevertheless, the intricacies of the TME also present novel opportunities for cancer treatment. The heightened presence of TAMs serves as a fresh avenue for exploration. The concept of bolstering the adaptive immune response by inhibiting TAM phagocytosis while enhancing its antigen‐presenting capabilities holds significant promise.^[^ ^263^ ^]^ Furthermore, for elderly and nonsurgically resectable patients, treatment primarily centered around chemoradiotherapy may only offer limited control over tumor recurrence and metastasis, along with extended survival. It remains of interest to observe whether the addition of efferocytosis inhibitors could potentially further extend survival outcomes. At present, a number of available clinical trial data prove that the single or combined use of efferocytosis inhibitors can achieve this desired result. However, drug safety is an issue worth tracking.^[^ ^226^, ^246^ ^]^ With the continuous elucidation of the molecular mechanisms associated with efferocytosis and the development of corresponding emerging drugs, we believe that more efferocytosis inhibitors with high efficacy and safety will emerge and eventually be granted clinical use.

## Conflict of Interest

The authors declare no conflict of interest.

## Author Contributions

**Gangxing Zhu**: data curation (lead); writing—original draft (lead). **Xinliang Wan**: data curation (lead). **Wanyin Wu**: supervision (lead). **Luyu Jia**: data curation (equal). **Xiaoyan Yu**: methodology (equal). **Handan Mo**: methodology (equal). **Xi Wang**: resources (equal). **Qichun Zhou**: validation (lead). **Qing Tang**: writing—review & editing (equal). **Sumei Wang**: conceptualization (lead); funding acquisition (lead); investigation (lead); supervision (lead); writing—review & editing (lead).
